# Association between thoracic adiposity and survival in non-metastatic breast cancer

**DOI:** 10.1186/s13058-025-02127-1

**Published:** 2025-10-14

**Authors:** Anlan Cao, Ijeamaka Anyene Fumagalli, Wendy Y. Chen, Adana A. M. Llanos, Charles P. Quesenberry, Bette J. Caan, Elizabeth M. Cespedes Feliciano

**Affiliations:** 1 Kaiser Permanente Division of Research, Kaiser Permanente Northern California, 4480 Hacienda Dr., Pleasanton, CA 94588 USA; 2 Department of Medical Oncology, Dana Farber Cancer Institute, Boston, MA USA; 3 Department of Epidemiology, Mailman School of Public Health, Columbia University Irving Medical Center, New York, NY USA; 4 Herbert Irving Comprehensive Cancer Center, Columbia University Irving Medical Center, New York, NY USA

**Keywords:** Body composition, Breast cancer, Chest CT, Cancer survival

## Abstract

**Background:**

Total adiposity measured by abdominal computed tomography (CT) at the third lumbar vertebrae (L3) has been associated with breast cancer survival, but most patients undergo chest CT. If adipose tissues at the thoracic level, including those surrounding the thoracic organs, are associated with survival, they could be used to inform care for significantly more breast cancer patients.

**Methods:**

We included 2127 individuals aged 18-< 90, diagnosed with stage II-III breast cancer at Kaiser Permanente Northern California (2005–2019). Cross-sectional areas of adiposity were quantified at the fourth thoracic vertebrae (T4) and L3. Using multivariable Cox models, we estimated hazard ratios (HRs) and 95% confidence intervals to compare the strength of association of T4-level versus L3-level adiposity with all‐cause and cause-specific mortality.

**Results:**

Participants were on average 56.2 years old at diagnosis. During an average follow-up of 8.2 years, 593 deaths occurred, with 100 from heart disease and 429 from breast cancer. Positive, moderate-to-strong correlations were observed between adiposity at T4 and L3. After adjusting for covariates including body mass index and muscle, higher intrathoracic and intermuscular adiposity at T4 were associated with increased all-cause (intrathoracic: HR = 1.35[1.06–1.72]; intermuscular: HR = 1.26[1.01–1.59]) and heart disease-specific mortality (intrathoracic: HR = 2.23[1.31–3.78]; intermuscular: HR = 2.25[1.37–3.68]). Greater subcutaneous adiposity at T4 showed a non-significant trend toward increased mortality (overall: HR = 1.24[0.95–1.61]; breast cancer-specific: 1.27[0.93–1.73]). These associations were not observed at L3.

**Conclusions:**

Despite strong correlations with L3, adiposity at T4 was significantly associated with overall and heart disease-specific mortality, while measurements at L3 were not, possibly due to the proximity of T4 to the breast tumor and heart.

**Supplementary Information:**

The online version contains supplementary material available at 10.1186/s13058-025-02127-1.

## Background

Breast cancer is the most commonly diagnosed cancer in women worldwide [1]. Thanks to advancements in treatment, individuals are living longer after a breast cancer diagnosis, contributing to a rapidly growing population of cancer survivors. About half of breast cancer patients are obese at the time of diagnosis [2], with a high burden of obesity-associated comorbidities, such as heart disease [3, 4], as well as an elevated risk of overall [5, 6] and cardiovascular disease-specific mortality [7]. Understanding the role of adiposity and its distribution in breast cancer prognosis could help inform targeted interventions to prolong survival.

The recent nutrition and physical activity guidelines for cancer survivors from the American Cancer Society [8] recommend that cancer survivors avoid obesity while highlighting that “more research is needed to evaluate the complex interactions between body composition and cancer progression, recurrence, site-specific mortality, and all-cause mortality.” This emphasis on body composition was echoed by a recent Lancet Commission’s diagnostic criteria for obesity [9], which underscored that “Body mass index (BMI) should be used only as a surrogate measure of health risk at a population level, rather than as an individual measure of health” as BMI does not differentiate between adipose and muscle tissue or account for the distribution of adiposity.

Clinical and epidemiologic studies often make opportunistic use of scans from diagnostic imaging, such as computed tomography (CT), to accurately quantify adiposity with minimal patient burden [10, 11]. Cross-sectional areas of adipose tissues at the third lumbar vertebra (L3) level are a common surrogate for whole-body adiposity and have been widely used in cancer research [12, 13]. While retrospective datasets often contain CT images suitable for body composition analysis, contemporary diagnostic imaging for breast cancer patients does not consistently include the L3 vertebral level: per the current National Comprehensive Cancer Network (NCCN) guidelines, chest CT is commonly used for radiation therapy planning, but typically does not extend to the abdomen, and CT imaging including the abdomen and pelvis is not recommended for asymptomatic patients with early-stage breast cancer [14]. Adipose tissue at the thoracic level from chest CT is close to both breasts and heart, and thus could be uniquely informative for both deaths associated with heart disease and breast cancer, two major causes of mortality among breast cancer patients [15].

The fourth thoracic vertebra (T4) level has been proposed as an alternative anatomic landmark for body composition assessment in oncology when L3 is unavailable and has been frequently used in lung cancer studies [16, 17]. A few studies assessed T4 body composition in breast cancer patients [18–20], but most had very small samples and did not evaluate the independent associations of these landmarks with breast cancer survival. It remains untested whether T4 adipose measurements are associated with breast cancer outcomes and the extent to which they can stand in for, or provide additional information, compared to measurements at L3.

To facilitate use of thoracic CT scans for prognostication and personalized care in breast cancer, we assessed the association between measures of adiposity at the T4 level and all-cause and cause-specific mortality in patients diagnosed with stage II-III breast cancer and compared the findings to measures of adiposity obtained at the L3 level.

## Methods

### Study population

We identified patients diagnosed with stage II-III primary invasive breast cancer at Kaiser Permanente Northern California (KPNC) between 2005 and 2019. Patients were eligible if they were 18-< 90 years old at time of diagnosis, underwent an abdominal or pelvic CT scan performed within six months of diagnosis but before chemotherapy and/or radiation, and had a plausible but not underweight BMI available (> 18.5 to < 68 kg/m^2^) within six months of CT date but prior to chemotherapy and/or radiation (N = 5194). We excluded scans that did not include L3 in the field of view (N = 1495). To facilitate comparison of the T4 and L3 measurements, we further excluded patients whose chest and abdomen were imaged in separate CT series (N = 1353). An additional 219 individuals were excluded due to the CT not including a continuous field of view from the T1-L5 vertebrae (N = 208), muscle being partially cut off in the image (N = 10), or arms in the field (N = 1). The final analytic cohort included 2127 patients whose breast cancer staging CT scans included both the thoracic and lumbar vertebrae (eFigure 1). The average time from diagnosis to CT scan was 1.01 ± 0.83 months.

### Body composition measurements

The majority of included patients were scanned in the feet-first supine position using GE Medical System scanners with a slice thickness of 5 mm. We utilized the Data Analysis Facilitation Suite (DAFS) [21] by Voronoi Health Analytics, Inc. (https://www.voronoihealthanalytics.com/) to obtain automated single-slice cross-sectional area (cm^2^) measurements of subcutaneous adipose tissue (SAT), intrathoracic adipose tissue (THAT, surrounding organs within the thoracic cavity), visceral adipose tissue (VAT, surrounding organs within the abdominal cavity), intermuscular adipose tissue (IMAT, adipose segmented between skeletal muscle tissues), and skeletal muscle (SKM). All images and segmentations were reviewed by a trained research assistant to exclude or flag imaging abnormalities based on predefined criteria.

**Fig. 1:**
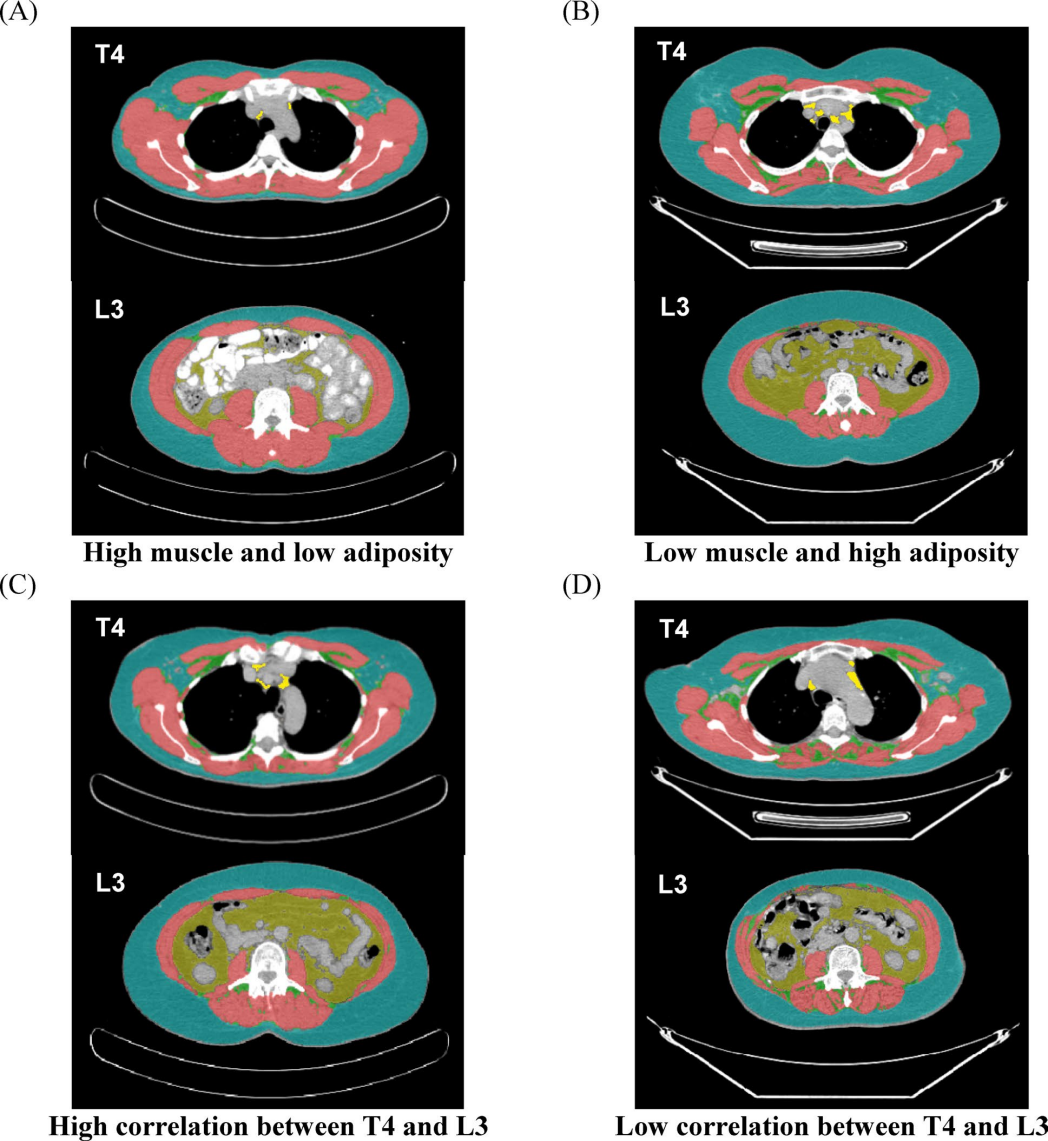
Representative computed tomography (CT) scans indicative of high and low correlations between the T4 and L3 levels. The figure shows example CT scans from patients with different body composition (high muscle, **A**; high adiposity: **B**), and patients with high (similar z-scores, **C**) or low correlations (disparate z-score values, **D**) for tissues at the T4 and L3 levels. The tissues are colored as subcutaneous adipose in blue, intrathoracic adipose in bright yellow, visceral adipose in mustard yellow, intermuscular adipose in green, and skeletal muscle in red. All individuals included had similar body mass index and were all in the category of 25-< 30 kg/m^2^

Of note, THAT included epicardial and paracardial adipose tissues (along the external surface of the pericardium). For SAT, IMAT and SKM, single-slice measurements were calculated at the mid-slices of T1 through L5 vertebrae. THAT exists only in the thoracic cavity and was obtained at the mid-slices of T1 through T10 vertebrae. Similarly, VAT is only in the abdominal cavity and was quantified at the mid-slices of T11 through L5 vertebrae (Fig. 1).

### Covariates

Demographic characteristics including age at diagnosis, self-reported race and ethnicity, and smoking history were collected from the KPNC electronic health records (EHRs) [22]. BMI closest to the scan (kg/m^2^) was calculated from weight and height measured at clinical visits. Breast cancer characteristics including American Joint Committee on Cancer (AJCC) stage, tumor grade, hormone receptor status (estrogen and progesterone receptor, ER/PR), human epidermal receptor-2 (HER2) status, and receipt of treatment (surgery, chemotherapy, radiation) were obtained from the KPNC Cancer Registry.

### Outcomes

We ascertained mortality from KPNC’s mortality files through the last available update (December 31, 2022), which incorporates internal data from the KPNC health system, and external linkages with mortality information from the State of California, the Social Security Administration and the National Death Index. We assigned heart disease or breast cancer mortality based on the immediate and underlying causes of death listed on the death certificate. Heart diseases were identified using the 10th revision of the International Classification of Diseases (ICD-10) codes recommended by the Centers for Disease Control and Prevention [23]. The ICD-10 codes are presented in eTable 1.

### Statistical analysis

To compare tissues with differing distributions, we standardized SAT, THAT, VAT, IMAT and SKM by subtracting the mean and dividing by the standard deviation (SD) to create tissue-specific z-scores. We then categorized these variables as > 1 SD lower of the mean, within 1 SD of the mean (reference), and > 1 SD higher of the mean. The linear correlation between thoracic and abdominal tissue area z‐scores were estimated using Pearson correlation coefficients.

Using Cox proportional hazards models, we estimated hazard ratios (HRs) and 95% confidence intervals (CIs) to compare the strength of association of T4-level versus L3-level measurements with all‐cause, heart disease-specific, and breast cancer-specific mortality. We reported aggregate *P*-values for each tissue from likelihood ratio tests. All models were adjusted for covariates including age at diagnosis, race and ethnicity (Non-Hispanic White, Hispanic, Black or African American, Asian or Pacific Islander, Other), stage (2/3), grade (well/moderately/poor differentiated/unknown), ER/PR (positive/negative/missing), HER2 (positive/negative/missing), treatment (yes/no: surgery, chemotherapy, radiation) and smoking (current/former/never/unknown). For each adipose tissue measure, we additionally adjusted for BMI (18.5-< 25, 25-< 30, 30 +) and SKM (within 1 SD, > 1SD lower, > 1SD higher). Person‐time was calculated as the years from the date of diagnosis to the date of death. If no event occurred, participants were censored at the end of the study period, 12/31/2022. We further conducted stratified analyses to explore potential effect modification by receptor status (ER/PR and HER2).

All statistical analyses were conducted using R Version 4.0.2. Statistical tests were 2-sided, with a *P* < 0.05 significance level.

## Results

During mean follow-up of 8.2 years (median: 7.9; max: 17.9), 593 deaths occurred, with 100 related to heart diseases, and 429 related to breast cancer. The participants were on average 56.2 years old at diagnosis. The cohort comprised 58.8% Non-Hispanic White, 7.9% Non-Hispanic Black, 13.0% Hispanic, 19.7% Asian or Pacific Islander, and 0.8% who reported being of another race and/or ethnicity (Table 1).

**Table 1:** Demographic and clinical characteristics of patients (N = 2127)

Characteristics	Patients, No. (%)^a^		
	Survived to end of follow-up (N = 1534)	Death from any cause (N = 593)	Overall (N = 2127)
Age at diagnosis, mean (SD)	54.2 (12.1)	61.3 (13.9)	56.2 (13.0)
Race and ethnicity			
Non-Hispanic White	884 (57.6)	366 (61.7)	1250 (58.8)
Non-Hispanic Black	111 (7.2)	56 (9.4)	167 (7.9)
Hispanic	208 (13.6)	68 (11.5)	276 (13.0)
Asian and Pacific Islander	325 (21.2)	93 (15.7)	418 (19.7)
Other	6 (0.4)	10 (1.7)	16 (0.8)
Stage			
Stage 2	970 (63.2)	243 (41.0)	1213 (57.0)
Stage 3	564 (36.8)	350 (59.0)	914 (43.0)
Grade			
Well differentiated	137 (8.9)	46 (7.8)	183 (8.6)
Moderately differentiated	618 (40.3)	232 (39.1)	850 (40.0)
Poor/undifferentiated	595 (38.8)	252 (42.5)	847 (39.8)
Unknown	184 (12.0)	63 (10.6)	247 (11.6)
ER/PR			
Either positive	1065 (69.4)	400 (67.5)	1465 (68.9)
Both negative	363 (23.7)	175 (29.5)	538 (25.3)
Missing	106 (6.9)	18 (3.0)	124 (5.8)
HER2			
Positive	421 (27.4)	89 (15.0)	510 (24.0)
Negative	1014 (66.1)	474 (79.9)	1488 (70.0)
Missing	99 (6.5)	30 (5.1)	129 (6.1)
Treatment			
Mastectomy	863 (56.3)	348 (58.7)	1211 (56.9)
Chemotherapy	1326 (86.4)	442 (74.5)	1768 (83.1)
Radiation	640 (41.7)	182 (30.7)	822 (38.6)
Smoke			
Current smoker	85 (5.5)	52 (8.8)	137 (6.4)
Former smoker	231 (15.1)	103 (17.4)	334 (15.7)
Never smoker	844 (55.0)	280 (47.2)	1124 (52.8)
Unknown	374 (24.4)	158 (26.6)	532 (25.0)
BMI (kg/m^2^)			
Normal (18.5 to < 25.0)	513 (33.4)	190 (32.0)	703 (33.1)
Overweight (25.0 to < 30.0)	493 (32.1)	179 (30.2)	672 (31.6)
Obese (30.0 +)	528 (34.4)	224 (37.8)	752 (35.4)
Comorbidities			
0	1143 (74.5)	364 (61.4)	1507 (70.9)
1–2	343 (22.4)	160 (27.0)	503 (23.6)
3 +	48 (3.1)	69 (11.6)	117 (5.5)

BMI, body mass index; ER/PR, estrogen receptors/progesterone receptors; HER2, human epidermal receptor 2; SD, standard deviation

^a^Percentages may not sum to 100% due to rounding

Most participants were diagnosed with stage II (57.0%), moderately or poorly differentiated tumors (79.8%) that were ER/PR + (68.9%) and HER2- (70.0%) (Table 1). Treatment rates were 56.9% for mastectomy, 83.1% for chemotherapy, and 38.6% for radiation. Over half of the participants were never smokers (52.8%), overweight or obese (67.0%), and had no comorbidity other than cancer at diagnosis (70.9%).

Those with more SAT at the T4 level were more likely to be non-Hispanic Black, current or former smokers, obese at diagnosis and had more comorbidities. They were also less likely to receive mastectomy (eTable 2). Baseline characteristics were similar between individuals included in the study and those excluded due to imaging-related issues (eTable 3).

### Body composition concordance

Area measurements of each body composition tissue depot at the T4 and L3 vertebrae are presented in eTable 4.

**Fig. 2:**
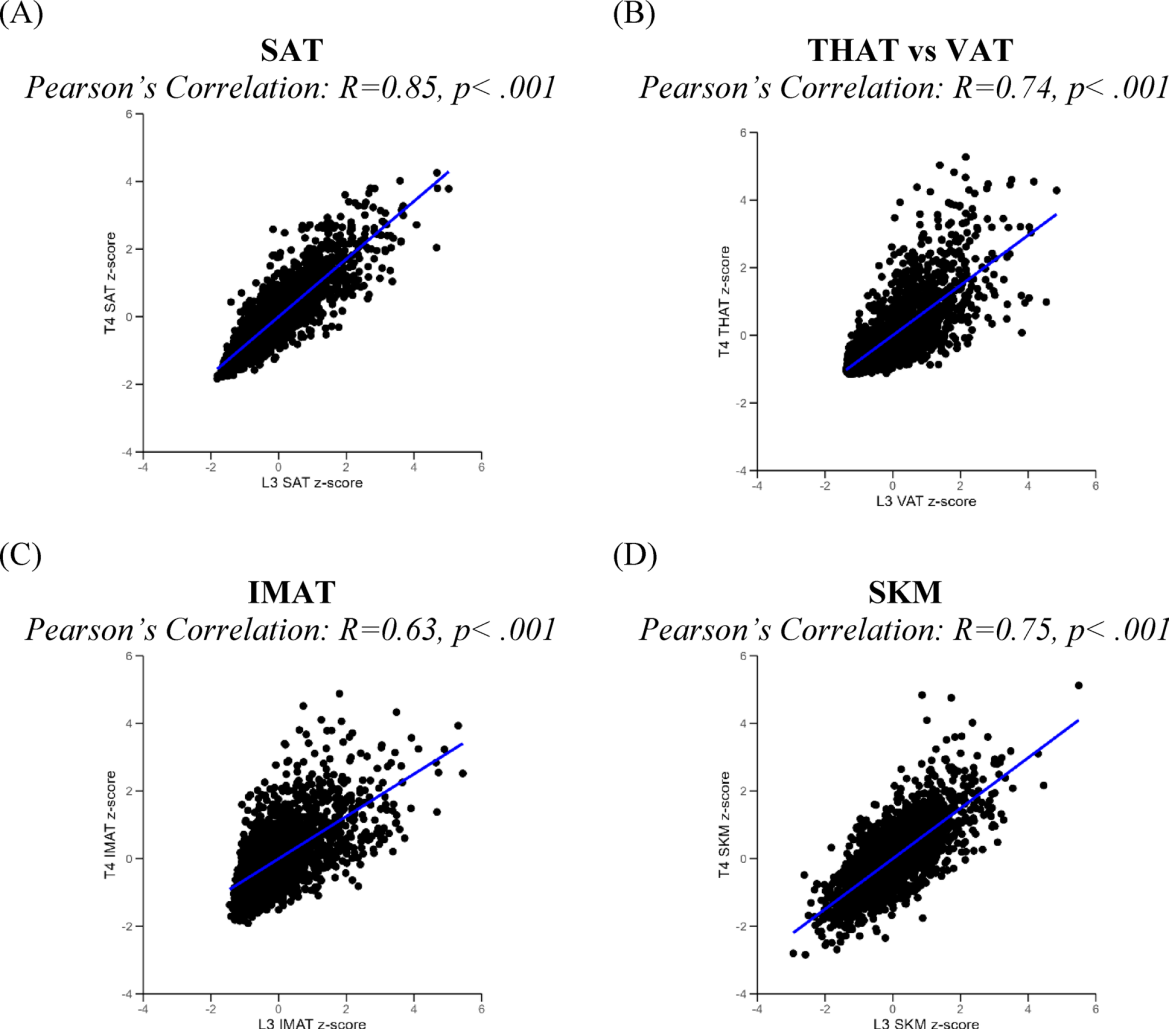
Scatter plots displaying the Pearson correlation coefficient. Comparing z-scores for body composition assessment from T4 vs L3 at diagnosis of breast cancer. IMAT, intermuscular adipose tissue; SAT, subcutaneous adipose tissue; SKM, skeletal muscle mass; THAT, intrathoracic adipose tissue; VAT, visceral adipose tissue

Overall, participants with greater adiposity at L3 also had greater adiposity at T4: all Pearson correlation coefficients were positive and ranged from moderate (r = 0.63 for IMAT) to strong (r = 0.85 for SAT) in magnitude, with 0.74 for THAT/VAT and 0.75 for SKM (eTable 5). However, there was substantial variation at the individual level as shown in Fig. 2. For example, a participant with average L3 IMAT (z-score = 0) could have T4 IMAT as much as 2 SD above the mean. Additionally, certain tissue depots are anatomically distinct but may be comparable, such as THAT around the organs within the thoracic cavity vs. VAT around the organs within the visceral cavity.

In general, participants had similar rankings when comparing SAT and SKM at L3 vs. T4 (eTable 6). About 20% of participants had inconsistent categorization across vertebrae (reflecting varied distribution of adiposity across the body), but none showed extreme discrepancies. For example, among individuals with an average L3 SAT (within 1 SD of the mean), 13% had T4 SAT that was either extremely high (> 1 SD above the mean) or extremely low (< 1 SD below the mean). However, none of the participants with extreme L3 SAT values exhibited the opposite extreme at T4, meaning none had a SAT > 1 SD higher at the L3 and < 1 SD lower at the T4 and vice versa.

### All-cause mortality

Table 2 presents the associations between body composition measurements at the T4 and L3 vertebral levels and all‐cause mortality. At T4, individuals with more THAT (HR = 1.27 [1.02–1.58] for > 1SD higher) and IMAT (HR = 1.24 [1.00–1.53] for > 1SD higher) had higher all-cause mortality compared to those with adipose tissue within 1 SD of the mean. The effect sizes remained similar when additionally adjusted for BMI and SKM (THAT: HR = 1.35 [1.06–1.72]; IMAT: HR = 1.26 [1.01–1.59]). There was also an indication that higher SAT was associated with all-cause mortality, though the association was not statistically significant (multivariable adjusted: HR = 1.24 [0.95–1.61]).

**Table 2:** Association between body composition and overall survival at T4 and L3 levels

Tissue depot	N (events)	Model 1^a^		Model 2^b^	
		HR (95% CI)	*P*-value^c^	HR (95% CI)	*P*-value^c^
T4 level					
SAT			0.24		0.12
Within 1SD	1499 (418)	1.00		1.00	
> 1SD lower	307 (74)	1.09 (0.85–1.40)		0.93 (0.70–1.24)	
> 1SD higher	321 (101)	1.21 (0.97–1.51)		1.24 (0.95–1.61)	
THAT			0.10		0.03
Within 1SD	1747 (478)	1.00		1.00	
> 1SD lower	97 (15)	0.87 (0.52–1.47)		0.78 (0.46–1.33)	
> 1SD higher	283 (100)	1.27 (1.02–1.58)		1.35 (1.06–1.72)	
IMAT			0.16		0.07
Within 1SD	1538 (424)	1.00		1.00	
> 1SD lower	294 (56)	1.01 (0.76–1.34)		0.93 (0.69–1.25)	
> 1SD higher	295 (113)	1.24 (1.00–1.53)		1.26 (1.01–1.59)	
SKM			0.57		–
Within 1SD	1482 (389)	1.00		–	
> 1SD lower	333 (121)	1.09 (0.87–1.35)		–	
> 1SD higher	312 (83)	1.11 (0.86–1.44)		–	
L3 level					
SAT			0.86		0.21
Within 1SD	1525 (430)	1.00		1.00	
> 1SD lower	288 (78)	1.05 (0.82–1.35)		0.95 (0.72–1.25)	
> 1SD higher	314 (85)	0.96 (0.76–1.23)		0.91 (0.69–1.19)	
VAT			0.66		0.22
Within 1SD	1035 (404)	1.00		1.00	
> 1SD lower	267 (74)	1.07 (0.83–1.38)		0.96 (0.73–1.28)	
> 1SD higher	232 (115)	1.09 (0.88–1.35)		1.08 (0.85–1.37)	
IMAT			0.05		0.03
Within 1SD	1622 (423)	1.00		1.00	
> 1SD lower	199 (43)	1.44 (1.03–2.02)		1.41 (1.00–1.98)	
> 1SD higher	306 (127)	1.17 (0.94–1.44)		1.18 (0.95–1.46)	
SKM			0.53		–
Within 1SD	1514 (407)	1.00		–	
> 1SD lower	294 (100)	1.01 (0.80–1.27)		–	
> 1SD higher	319 (86)	1.15 (0.90–1.47)		–	

BMI, body mass index; CI, confidence interval; ER/PR, estrogen receptors/progesterone receptors; HER2, human epidermal receptor 2; HR, hazard ratio; IMAT, intermuscular adipose tissue; SAT, subcutaneous adipose tissue; SD, standard deviation; SKM, skeletal muscle mass; THAT, intrathoracic adipose tissue; VAT, visceral adipose tissue

^a^Adjusted for age at diagnosis, race and ethnicity, stage, grade, ER/PR, HER2, treatment (surgery, chemo, radiation), and smoking

^b^Additionally adjusted for BMI and SKM

^c^Aggregate *P*-value from likelihood ratio test

At L3, lower IMAT was associated with higher all-cause mortality (fully adjusted: HR = 1.41 [1.00–1.98]). However, no association was found between SAT or VAT and overall survival at L3, and the associations of higher SAT, VAT and IMAT differed from those observed at T4 (at L3, SAT: HR = 0.91 [0.69–1.19]; VAT: HR = 1.08 [0.85–1.37]; IMAT: HR = 1.18 [0.95–1.46] for > 1 SD higher vs. within 1 SD).

Associations were similar when comparing patient subgroups based on ER/PR (positive vs. negative) or HER2 (positive vs. negative) receptor status (eFigures  2 and  3).

### Cause-specific mortality

Table 3 presents the results for cause-specific mortality. After adjusting for all covariates including BMI and SKM, greater THAT and IMAT at T4 were significantly associated with worse heart disease-specific mortality (THAT: HR = 2.23 [1.31–3.78]; IMAT: HR = 2.25 [1.37–3.68] for > 1SD higher vs. within 1 SD). No association was found between L3 adipose tissues and heart disease-specific mortality.

**Table 3:** Association between body composition and cause-specific survival at T4 and L3 levels

Tissue depot	Heart disease-specific mortality					Breast cancer-specific mortality				
	N (events)	Model 1^a^		Model 2^b^		N (events)	Model 1^a^		Model 2^b^	
		HR (95% CI)	P^c^	HR (95% CI)	P^c^		HR (95% CI)	P^c^	HR (95% CI)	P^c^
T4 level										
SAT			0.11		0.11			0.45		0.09
Within 1SD	1499 (63)	1.00		1.00		1499 (301)	1.00		1.00	
> 1SD lower	307 (14)	1.45 (0.80–2.61)		1.30 (0.63–2.67)		307 (54)	1.03 (0.77–1.39)		0.83 (0.60–1.17)	
> 1SD higher	321 (23)	1.66 (1.01–2.73)		1.28 (0.72–2.25)		321 (74)	1.19 (0.91–1.54)		1.27 (0.93–1.73)	
THAT			0.01		< 0.001			0.82		0.22
Within 1SD	1747 (71)	1.00		1.00		1747 (355)	1.00		1.00	
> 1SD lower	97 (2)	0.76 (0.18–3.17)		0.69 (0.16–2.94)		97 (13)	0.89 (0.50–1.56)		0.80 (0.45–1.42)	
> 1SD higher	283 (27)	2.20 (1.39–3.48)		2.23 (1.31–3.78)		283 (61)	1.07 (0.81–1.40)		1.11 (0.82–1.51)	
IMAT			< 0.001		< 0.001			0.91		0.28
Within 1SD	1538 (59)	1.00		1.00		1538 (313)	1.00		1.00	
> 1SD lower	294 (11)	1.54 (0.79–2.98)		1.52 (0.75–3.05)		294 (48)	1.01 (0.74–1.39)		0.93 (0.67–1.30)	
> 1SD higher	295 (30)	2.42 (1.53–3.82)		2.25 (1.37–3.68)		295 (68)	1.06 (0.81–1.39)		1.08 (0.81–1.43)	
SKM			0.19		–			0.59		–
Within 1SD	1482 (59)	1.00		–		1482 (288)	1.00		–	
> 1SD lower	333 (21)	1.33 (0.78–2.29)		–		333 (73)	1.02 (0.78–1.34)		–	
> 1SD higher	312 (20)	1.59 (0.91–2.79)		–		312 (68)	1.16 (0.88–1.55)		–	
L3 level										
SAT			0.24		0.06			0.91		0.18
Within 1SD	1525 (64)	1.00		1.00		1525 (312)	1.00		1.00	
> 1SD lower	288 (17)	1.58 (0.92–2.74)		1.68 (0.86–3.27)		288 (54)	0.99 (0.74–1.33)		0.85 (0.61–1.18)	
> 1SD higher	314 (19)	1.25 (0.73–2.15)		0.88 (0.49–1.59)		314 (63)	0.94 (0.71–1.25)		0.92 (0.67–1.27)	
VAT			0.26		0.11			0.74		0.28
Within 1SD	1439 (62)	1.00		1.00		1439 (292)	1.00		1.00	
> 1SD lower	341 (13)	1.30 (0.70–2.41)		1.25 (0.62–2.53)		341 (63)	1.11 (0.84–1.48)		1.00 (0.73–1.37)	
> 1SD higher	347 (25)	1.47 (0.91–2.36)		1.22 (0.71–2.08)		347 (74)	0.99 (0.77–1.29)		0.98 (0.73–1.31)	
IMAT			0.14		0.04			0.16		0.08
Within 1SD	1622 (66)	1.00		1.00		1622 (313)	1.00		1.00	
> 1SD lower	199 (8)	1.91 (0.85–4.27)		1.99 (0.87–4.55)		199 (39)	1.39 (0.97–1.99)		1.34 (0.93–1.93)	
> 1SD higher	306 (26)	1.41 (0.86–2.29)		1.28 (0.77–2.12)		306 (77)	1.11 (0.85–1.45)		1.13 (0.86–1.48)	
SKM			0.54		–			0.76		–
Within 1SD	1514 (66)	1.00		–		1514 (299)	1.00		–	
> 1SD lower	294 (16)	1.09 (0.61–1.94)		–		294 (63)	0.98 (0.73–1.31)		–	
> 1SD higher	319 (18)	1.38 (0.79–2.4)		–		319 (67)	1.11 (0.84–1.46)		–	

BMI, body mass index; CI, confidence interval; ER/PR, estrogen receptors/progesterone receptors; HER2, human epidermal receptor 2; HR, hazard ratio; IMAT, intermuscular adipose tissue; SAT, subcutaneous adipose tissue; SD, standard deviation; SKM, skeletal muscle mass; THAT, intrathoracic adipose tissue; VAT, visceral adipose tissue

^a^Adjusted for age at diagnosis, race and ethnicity, stage, grade, ER/PR, HER2, treatment (surgery, chemo, radiation), and smoking

^b^Additionally adjusted for BMI and SKM

^c^Aggregate *P*-value from likelihood ratio test

For breast cancer-specific mortality, consistent with all-cause mortality, there was a suggestive but non-significant association between higher SAT at T4 and increased breast cancer-specific mortality (HR = 1.27 [0.93–1.73]). No other body composition measures were associated with breast cancer-specific mortality.

## Discussion

Despite the strong, positive correlation between body composition measured at T4 and L3, we observed different associations between adipose tissues measured at different vertebrae and mortality in patients diagnosed with breast cancer. At T4, increased adipose tissue around the thoracic organs (THAT) and adipose tissue between the thoracic muscles (IMAT) were significantly associated with higher all-cause and heart disease-specific mortality, with suggestive but non-significant associations indicated between SAT and all-cause and breast cancer-specific mortality. However, none of the associations held at L3 in our cohort.

This is the first large-scale study to assess adipose tissue in the thoracic region in relation to breast cancer prognosis. Similar to our study, two smaller studies examined the concordance of body composition measurements at T4 and L3 in breast cancer patients and reported strong positive correlations between T4 and L3 tissues [18, 20]. Most prior investigations of adiposity and breast cancer prognosis used imaging-measured adipose tissue areas at abdominal level [24, 25]. Although a small study of Asian individuals with breast cancer found that higher upper VAT volume, rather than cross-sectional area, was associated with poorer distant disease-free survival in patients who underwent chemotherapy [26], the majority of studies found no statistically significant association between higher SAT [27–30], VAT [27–31] or IMAT [30] at L3 and overall survival in breast cancer patients, consistent with our findings at L3. Meanwhile, higher BMI and total adiposity measured by CT at diagnosis has been associated with worse survival in breast cancer patients [5, 6, 32]. Our findings suggest that, independent of BMI, thoracic adiposity may have a stronger influence on breast cancer prognosis than abdominal adiposity.

Given the importance of non-cancer mortality among breast cancer survivors, we examined specific causes of death and found that higher THAT and IMAT at T4 were significantly associated with heart disease-specific mortality. In contrast, neither higher VAT nor higher IMAT at L3 were related to heart disease-specific mortality. THAT may be more relevant to cardiovascular morbidity and mortality, as it includes all adipose tissue surrounding the heart, such as epicardial and pericardial adipose tissue, both of which have been associated with risk of cardiotoxicities in breast cancer patients [33, 34]. Regarding IMAT, a recent study reported that increased IMAT at T12 was associated with a higher incidence of heart events in a population without cancer [35], which aligns with another study that found IMAT in the thigh muscle to be independently related to heart failure [36]. In the breast cancer-specific mortality analysis, SAT at T4 level appeared to be relevant. Increased adipocytes near the breast can lead to higher local estrogen concentrations [37, 38], which have been associated with an increased risk of breast cancer development [39] and possibly disease progression.

This large-scale study demonstrated that adiposity at thoracic levels is associated with mortality after breast cancer, reinforcing the utility of chest CT for body composition assessment in breast cancer patients. Our findings align with research in lung cancer in which adipose tissues such as IMAT measured at thoracic vertebrae [17] have been linked to worse overall survival [40]. When available, thoracic body composition should be taken into consideration when building risk prediction models for treatment selection and tailored supportive care for breast cancer patients.

This study is not without limitations. Our cohort was restricted to stage II/III patients, as stage I breast cancer patients do not routinely receive CT scans. However, our prior study found that the characteristics of women who did and did not undergo CT were similar across stage I-III patients [41], indicating that our findings may be generalizable. Additionally, surveillance CT scans are not routinely performed in breast cancer patients, precluding the assessment of longitudinal changes in body composition relevant to the progression of breast cancer and heart disease. Lastly, the number of heart disease-related mortality events was relatively small; future studies with a larger number of events are needed to verify our findings.

## Conclusions

In summary, we assessed the association between adipose tissues measured using chest CT scans and survival in a large sample of stage II-III breast cancer patients from a community oncology setting. Although highly correlated with L3, adipose tissue measurements at the T4 level were associated with overall mortality as well as death from heart disease, while measurements at L3 were not, possibly due to the proximity of T4 tissues to the breast tumor and heart. Future studies should incorporate thoracic CT scans when available, either independently or alongside L3 level measurements, as they can help elucidate the role of adiposity in breast cancer survival and potentially enhance prediction of cancer prognosis. Larger studies are needed to translate these findings into clinical risk stratification tools to improve breast cancer outcomes and inform lifestyle interventions during treatment.

## Supplementary Information

Below is the link to the electronic supplementary material.

Supplementary Material 1.

## Data Availability

The datasets generated and/or analyzed during the current study are not publicly available because they contain protected health information (PHI) of the participants, but are available from the corresponding author on reasonable request with appropriate ethics approvals and data sharing agreements.
