# Supplementary material for: Association between thoracic adiposity and survival in non-metastatic breast cancer
Source: Breast Cancer Res. 2025 Oct 14;27:176. doi: 10.1186/s13058-025-02127-1 (PMC12523217; doi:10.1186/s13058-025-02127-1)
Supplement: Supplementary file 1 — Supplementary Material 1. [file 13058_2025_2127_MOESM1_ESM.docx]

**Supplementary information**

Prognostic Value of Thoracic Adiposity in Non-Metastatic Breast Cancer Survival

**eFigure 1.** Cohort selection diagram

**eFigure 2.** Association between body composition and overall survival stratified by ER/PR status

**eFigure 3.** Association between body composition and overall survival stratified by HER2 status

**eTable 1.** ICD-10 codes for cause-specific mortality

**eTable 2.** Demographic and clinical characteristics of patients by T4 SAT (N=2,127)

**eTable 3.** Demographic and clinical characteristics of patients by selection (N=5,194)

**eTable 4.** Body composition by SD group and vertebra level

**eTable 5.** Correlation of tissue areas measured at thoracic and L3 levels

**eTable 6.** Distribution of patients according to T4 and L3 body composition categories

KPNC members diagnosed with breast cancer between January 1, 2005 and December 31, 2019, who are women and aged between 18 and 90

(n = 51,415)

Women who are stage II or stage III defined using AJCC Cancer Staging

(n = 16,604)

Women whose first primary cancer is invasive breast cancer or DCIS with subsequent invasive breast cancer

(n = 14,425)

Has whole body PET/CT, abdominal CT, or pelvic CT completed from 6 months prior to diagnosis date to before chemo/radiation, or within 6 months if no chemo/radiation

(n = 5,526)

Had a valid BMI (>18.5 to <68) available within 12-month window of the scan date, but prior to chemo/radiation if completed, and maintained continuous enrollment that overlaps diagnosis date

(n = 5,194)

Scans did not include L3 in the field of view

(n=1,495)

Chest and abdomen in separate CT series

(n=1,353)

No continuous field of view from T1-L5

(n=208)

Scan contained all scans from T1 to L5

(n = 2,138)

No muscle cut out or

arms in field of view at T4

(n = 2,127)

**eFigure 1.** Cohort selection diagram

| **a) T4 level** | **b) L3 level** |
| --- | --- |
| **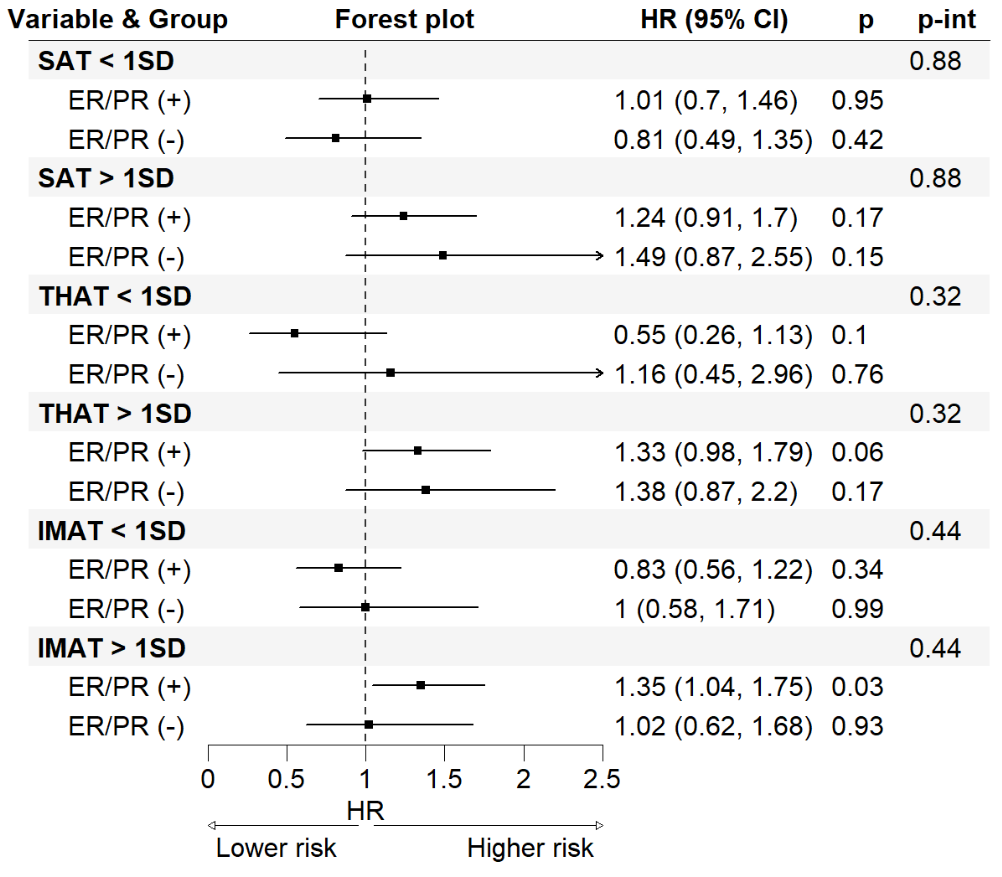** | **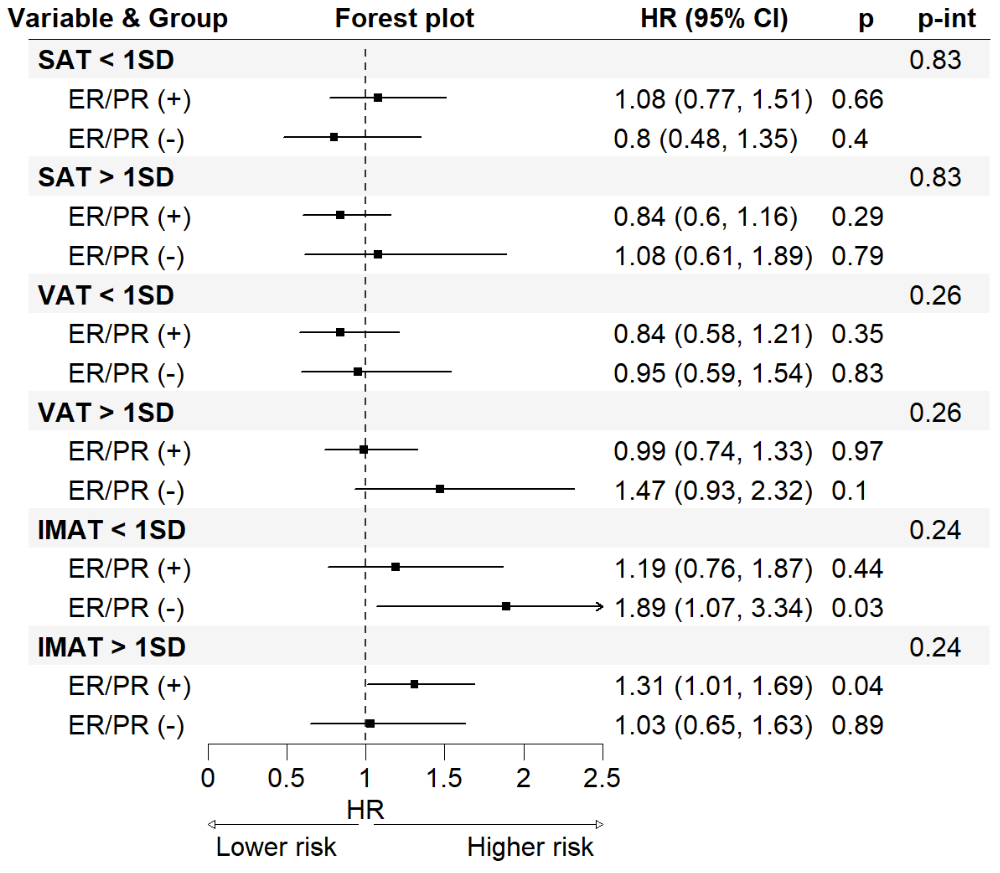** |

**eFigure 2.** Association between body composition and overall survival stratified by ER/PR status

Results adjusted for age at diagnosis, race and ethnicity, stage, grade, HER2, treatment (surgery, chemo, radiation), smoking, BMI and SKM. P-int are p for interactions from likelihood ratio test.

Abbreviation: BMI: Body mass index; CI: Confidence interval; ER/PR: Estrogen receptors/Progesterone receptors; HER2: Human epidermal receptor 2; HR: Hazard ratio; IMAT: Intermuscular adipose tissue; SAT: Subcutaneous adipose tissue; SD: Standard deviation; SKM: Skeletal muscle mass; THAT: Intrathoracic adipose tissue; VAT: Visceral adipose tissue.

| **a) T4 level** | **b) L3 level** |
| --- | --- |
| **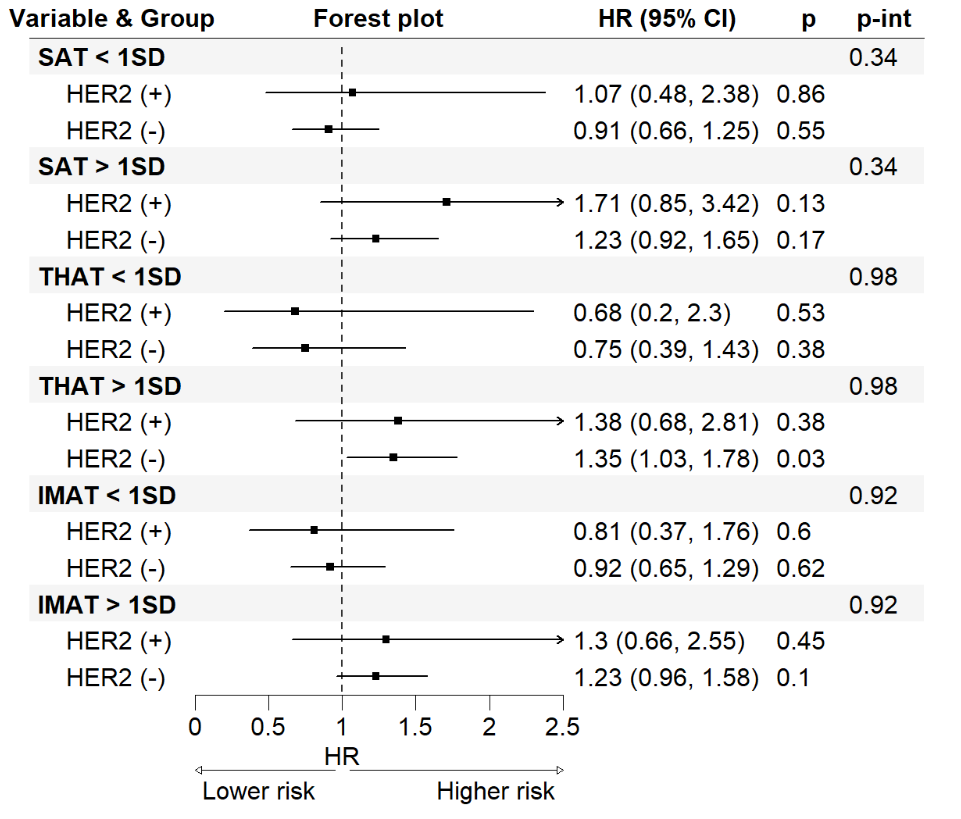** | **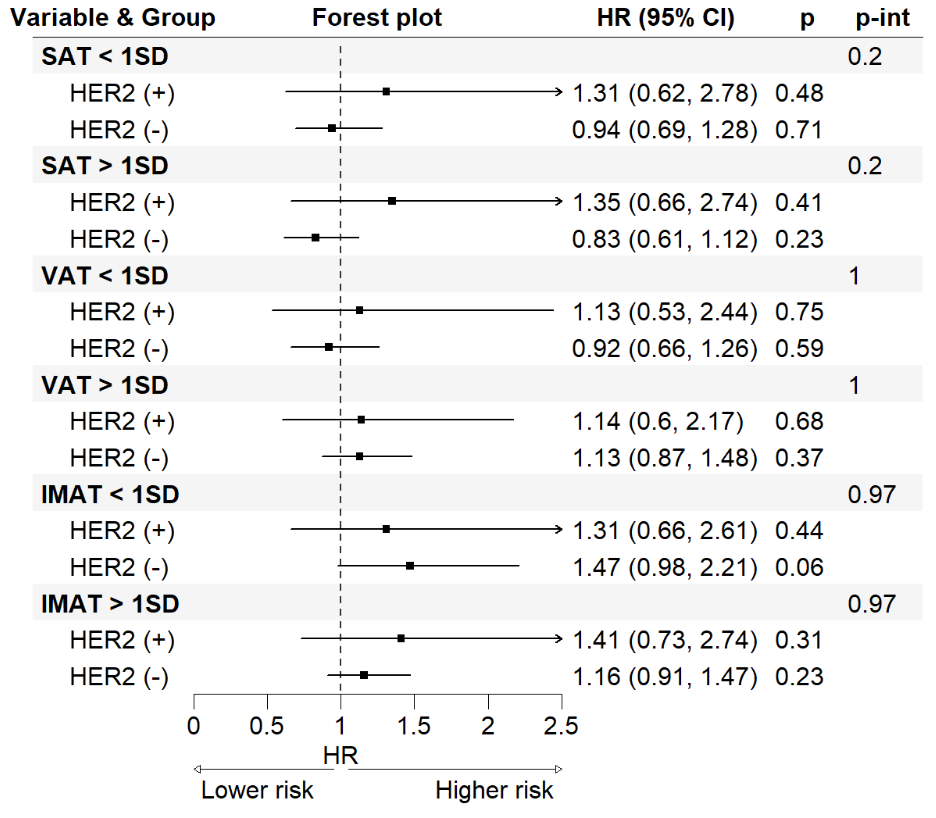** |

**eFigure 3.** Association between body composition and overall survival stratified by HER2 status

Results adjusted for age at diagnosis, race and ethnicity, stage, grade, ER/PR, treatment (surgery, chemo, radiation), smoking, BMI and SKM. P-int are p for interactions from likelihood ratio test.

Abbreviation: BMI: Body mass index; CI: Confidence interval; ER/PR: Estrogen receptors/Progesterone receptors; HER2: Human epidermal receptor 2; HR: Hazard ratio; IMAT: Intermuscular adipose tissue; SAT: Subcutaneous adipose tissue; SD: Standard deviation; SKM: Skeletal muscle mass; THAT: Intrathoracic adipose tissue; VAT: Visceral adipose tissue.

**eTable 1.** ICD-10 codes for cause-specific mortality

|  | **ICD-10 codes** |
| --- | --- |
| **Heart disease-specific death** | I00, I01, I02, I05, I06, I07, I08, I09, I11, I13, I2, I3, I4, I50, I51, I52 |
| **Breast cancer-specific death** | C50.0, C50.8, C50.9 |

Abbreviation: ICD: International Classification of Diseases

**eTable 2.** Demographic and clinical characteristics of patients by T4 SAT (N=2,127)

| **Characteristics** | **Patients, No. (%)^a^** | | |
| --- | --- | --- | --- |
|  | **>1SD lower**  **(N=307)** | **Within 1 SD**  **(N=1,499)** | **>1SD higher**  **(N=321)** |
| **Age at diagnosis, mean (SD)** | 50.2 (14.2) | 57.3 (12.7) | 56.6 (11.6) |
| **Race and Ethnicity** |  |  |  |
| Non-Hispanic White | 177 (57.7) | 869 (58.0) | 204 (63.6) |
| Non-Hispanic Black | 12 (3.9) | 106 (7.1) | 49 (15.3) |
| Hispanic | 29 (9.4) | 200 (13.3) | 47 (14.6) |
| Asian and Pacific Islander | 86 (28.0) | 315 (21.0) | 17 (5.3) |
| Other | 3 (1.0) | 9 (0.6) | 4 (1.2) |
| **Stage** |  |  |  |
| Stage 2 | 198 (64.5) | 836 (55.8) | 179 (55.8) |
| Stage 3 | 109 (35.5) | 663 (44.2) | 142 (44.2) |
| **Grade** |  |  |  |
| Well differentiated | 29 (9.4) | 136 (9.1) | 18 (5.6) |
| Moderately differentiated | 134 (43.6) | 585 (39.0) | 131 (40.8) |
| Poor/undifferentiated | 111 (36.2) | 610 (40.7) | 126 (39.3) |
| Unknown | 33 (10.7) | 168 (11.2) | 46 (14.3) |
| **ER/PR** |  |  |  |
| Either positive | 219 (71.3) | 1017 (67.8) | 229 (71.3) |
| Both negative | 72 (23.5) | 397 (26.5) | 69 (21.5) |
| Missing | 16 (5.2) | 85 (5.7) | 23 (7.2) |
| **HER2** |  |  |  |
| Positive | 85 (27.7) | 354 (23.6) | 71 (22.1) |
| Negative | 206 (67.1) | 1055 (70.4) | 227 (70.7) |
| Missing | 16 (5.2) | 90 (6.0) | 23 (7.2) |
| **Treatment** |  |  |  |
| Mastectomy | 221 (72.0) | 839 (56.0) | 151 (47.0) |
| Chemotherapy | 256 (83.4) | 1230 (82.1) | 282 (87.9) |
| Radiation | 107 (34.9) | 573 (38.2) | 142 (44.2) |
| **Smoke** |  |  |  |
| Current smoker | 21 (6.8) | 89 (5.9) | 27 (8.4) |
| Former smoker | 32 (10.4) | 231 (15.4) | 71 (22.1) |
| Never smoker | 166 (54.1) | 804 (53.6) | 154 (48.0) |
| Unknown | 88 (28.7) | 375 (25.0) | 69 (21.5) |
| **BMI (kg/m^2^)** |  |  |  |
| Normal (18.5 to <25.0) | 291 (94.8) | 412 (27.5) | 0 (0.0) |
| Overweight (25.0 to <30.0) | 16 (5.2) | 649 (43.3) | 7 (2.2) |
| Obese (30.0+) | 0 (0.0) | 438 (29.2) | 314 (97.8) |
| **Comorbidities** |  |  |  |
| 0 | 259 (84.4) | 1068 (71.2) | 180 (56.1) |
| 1-2 | 43 (14.0) | 352 (23.5) | 108 (33.6) |
| 3+ | 5 (1.6) | 79 (5.3) | 33 (10.3) |

Abbreviation: BMI: Body mass index; ER/PR: Estrogen receptors/Progesterone receptors; HER2: Human epidermal receptor 2; SD: Standard deviation

^a^Percentages may not sum to 100% due to rounding.

**eTable 3.** Demographic and clinical characteristics of patients by selection (N=5,194)

| **Characteristics** | **Patients, No. (%)^a^** | | |
| --- | --- | --- | --- |
|  | **Excluded^b^**  **(N=3,067)** | **Included**  **(N=2,127)** | **Overall**  **(N=5,194)** |
| **Age at diagnosis, mean (SD)** | 57.3 (13.6) | 56.2 (13.0) | 56.8 (13.4) |
| **Race and Ethnicity** |  |  |  |
| Non-Hispanic White | 1808 (59.0) | 1250 (58.8) | 3058 (58.9) |
| Non-Hispanic Black | 264 (8.6) | 167 (7.9) | 431 (8.3) |
| Hispanic | 451 (14.7) | 276 (13.0) | 727 (14.0) |
| Asian and Pacific Islander | 515 (16.8) | 418 (19.7) | 933 (18.0) |
| Other | 29 (0.9) | 16 (0.8) | 45 (0.9) |
| **Stage** |  |  |  |
| Stage 2 | 1347 (43.9) | 914 (43.0) | 2261 (43.5) |
| Stage 3 | 1720 (56.1) | 1213 (57.0) | 2933 (56.5) |
| **Grade** |  |  |  |
| Well differentiated | 297 (9.7) | 183 (8.6) | 480 (9.2) |
| Moderately differentiated | 1051 (34.3) | 850 (40.0) | 1901 (36.6) |
| Poor/undifferentiated | 1100 (35.9) | 847 (39.8) | 1947 (37.5) |
| Unknown | 619 (20.2) | 247 (11.6) | 866 (16.7) |
| **ER/PR** |  |  |  |
| Either positive | 1974 (64.4) | 1465 (68.9) | 3439 (66.2) |
| Both negative | 631 (20.6) | 538 (25.3) | 1169 (22.5) |
| Missing | 462 (15.1) | 124 (5.8) | 586 (11.3) |
| **HER2** |  |  |  |
| Positive | 618 (20.2) | 510 (24.0) | 1128 (21.7) |
| Negative | 1967 (64.1) | 1488 (70.0) | 3455 (66.5) |
| Missing | 482 (15.7) | 129 (6.1) | 611 (11.8) |
| **Treatment** |  |  |  |
| Mastectomy | 1876 (61.2) | 1211 (56.9) | 3087 (59.4) |
| Chemotherapy | 2352 (76.7) | 1768 (83.1) | 4120 (79.3) |
| Radiation | 1182 (38.5) | 822 (38.6) | 2004 (38.6) |
| **Smoke** |  |  |  |
| Current smoker | 216 (7.0) | 137 (6.4) | 353 (6.8) |
| Former smoker | 413 (13.5) | 334 (15.7) | 747 (14.4) |
| Never smoker | 1350 (44.0) | 1124 (52.8) | 2474 (47.6) |
| Unknown | 1088 (35.5) | 532 (25.0) | 1620 (31.2) |
| **BMI (kg/m^2^)** |  |  |  |
| Normal (18.5 to <25.0) | 977 (31.9) | 703 (33.1) | 1680 (32.3) |
| Overweight (25.0 to <30.0) | 995 (32.4) | 672 (31.6) | 1667 (32.1) |
| Obese (30.0+) | 1095 (35.7) | 752 (35.4) | 1847 (35.6) |
| **Comorbidities** |  |  |  |
| 0 | 1973 (64.3) | 1507 (70.9) | 3480 (67.0) |
| 1-2 | 816 (26.6) | 503 (23.6) | 1319 (25.4) |
| 3+ | 278 (9.1) | 117 (5.5) | 395 (7.6) |

Abbreviation: BMI: Body mass index; ER/PR: Estrogen receptors/Progesterone receptors; HER2: Human epidermal receptor 2; SD: Standard deviation

^a^Percentages may not sum to 100% due to rounding.

^b^Excluded due to imaging-related issues: not having L3 in the field of view, chest and abdominal CT scans acquired in the separate series, no continuous T1 to L5 scans, muscle group cut off at T4 or arms in the field of view.

**eTable 4.** Body composition by SD group and vertebra level

| **Tissue depot** | **> 1SD lower^a^** | **Within 1SD^a^** | **> 1SD higher^a^** |
| --- | --- | --- | --- |
| **T4 level** | | | |
| **SAT** |  |  |  |
| N (%) | 307 (14.43) | 1499 (70.47) | 321 (15.09) |
| Area (cm^2^), mean (SD) | 81.41 (21.70) | 212.10 (61.68) | 429.75 (76.13) |
| **THAT** |  |  |  |
| N (%) | 97 (4.56) | 1747 (82.13) | 283 (13.31) |
| Area (cm^2^), mean (SD) | 0.32 (0.18) | 3.95 (2.38) | 14.5 (4.26) |
| **IMAT** |  |  |  |
| N (%) | 294 (13.82) | 1538 (72.31) | 295 (13.87) |
| Area (cm^2^), mean (SD) | 8.64 (2.06) | 20.48 (5.45) | 40.63 (8.05) |
| **SKM** |  |  |  |
| N (%) | 333 (15.66) | 1482 (69.68) | 312 (14.67) |
| Area (cm^2^), mean (SD) | 120.06 (9.43) | 156.42 (14.33) | 202.26 (18.22) |
| **L3 level** | | | |
| **SAT** |  |  |  |
| N (%) | 288 (13.54) | 1525 (71.70) | 314 (14.76) |
| Area (cm^2^), mean (SD) | 100.32 (24.11) | 243.47 (69.96) | 495.95 (94.91) |
| **VAT** |  |  |  |
| N (%) | 341 (16.03) | 1439 (67.65) | 347 (16.31) |
| Area (cm^2^), mean (SD) | 18.74 (7.91) | 101.02 (43.59) | 253.43 (56.15) |
| **IMAT** |  |  |  |
| N (%) | 199 (9.36) | 1622 (76.26) | 306 (14.39) |
| Area (cm^2^), mean (SD) | 3.1 (0.65) | 9.28 (3.63) | 23.88 (5.68) |
| **SKM** |  |  |  |
| N (%) | 294 (13.82) | 1514 (70.18) | 319 (15.00) |
| Area (cm^2^), mean (SD) | 84.47 (6.98) | 111.91 (11.11) | 147.95 (13.28) |

Abbreviation: IMAT: Intermuscular adipose tissue; SAT: Subcutaneous adipose tissue; SD: Standard deviation; SKM: Skeletal muscle mass; THAT: Intrathoracic adipose tissue; VAT: Visceral adipose tissue

^a^Z-score (mean=0, sd=1) based groups

**eTable 5.** Correlation of tissue areas measured at thoracic and L3 levels

| **Thoracic Level** | **Pearson correlation coefficient** | | | |
| --- | --- | --- | --- | --- |
|  | **SAT** | **THAT/VAT^a^** | **IMAT** | **SKM** |
| **T1** | 0.82 | 0.2 | 0.49 | 0.71 |
| **T2** | 0.84 | 0.7 | 0.55 | 0.75 |
| **T3** | 0.85 | 0.74 | 0.58 | 0.75 |
| **T4** | 0.85 | 0.74 | 0.63 | 0.75 |
| **T5** | 0.85 | 0.79 | 0.65 | 0.7 |
| **T6** | 0.86 | 0.76 | 0.61 | 0.64 |
| **T7** | 0.86 | 0.75 | 0.57 | 0.59 |
| **T8** | 0.85 | 0.72 | 0.58 | 0.64 |
| **T9** | 0.84 | 0.75 | 0.6 | 0.72 |
| **T10** | 0.84 | 0.61 | 0.63 | 0.77 |
| **T11** | 0.89 | 0.75 | 0.68 | 0.8 |
| **T12** | 0.92 | 0.84 | 0.72 | 0.84 |

Abbreviation: IMAT: Intermuscular adipose tissue; SAT: Subcutaneous adipose tissue; SKM: Skeletal muscle mass; THAT: Intrathoracic adipose tissue; VAT: Visceral adipose tissue

^a^Correlation between T1-T10 THAT and L3 VAT; or T11-T12 VAT and L3 VAT.

**eTable 6.** Distribution of patients according to T4 and L3 body composition categories

|  | | **Patients, No. (%)** | | |
| --- | --- | --- | --- | --- |
| **SAT** | | **T4** | | |
|  |  | **> 1SD lower** | **Within 1SD** | **> 1SD higher** |
| **L3** | **> 1SD lower** | 202 (9.5) | 86 (4) | 0 (0) |
|  | **Within 1SD** | 105 (4.9) | 1321 (62.1) | 99 (4.7) |
|  | **> 1SD higher** | 0 (0) | 92 (4.3) | 222 (10.4) |
| **SKM** | | **T4** | | |
|  |  | **> 1SD lower** | **Within 1SD** | **> 1SD higher** |
| **L3** | **> 1SD lower** | 170 (8) | 124 (5.8) | 0 (0) |
|  | **Within 1SD** | 163 (7.7) | 1230 (57.8) | 121 (5.7) |
|  | **> 1SD higher** | 0 (0) | 128 (6) | 191 (9) |

Abbreviation: SAT: Subcutaneous adipose tissue; SD: Standard deviation; SKM: Skeletal muscle mass
